# ITS amplicon sequencing revealed that rare taxa of tea rhizosphere fungi are closely related to the environment and provide feedback on tea tree diseases

**DOI:** 10.1128/spectrum.01889-24

**Published:** 2024-11-29

**Authors:** Yuanqi Zhao, Weiwei Ran, Wenming Xu, Yuehua Song

**Affiliations:** 1 School of Karst Science, Guizhou Normal University 12686 , Guiyang, China; 2 State Engineering Technology Institute for Karst Desertification Control, Guiyang, China; Dominican University New York, Orangeburg, New York, USA

**Keywords:** rhizosphere fungi, rare taxa, cooccurrence network, individual soil function, tea tree, karst

## Abstract

The rhizospheres of plants and soil microorganisms are intricately interconnected. Tea trees are cultivated extensively on the karst plateau of Guizhou Province, China; however, the understanding of the interactions among fungal communities, community taxa, and diseases impacting tea tree in the soil rhizosphere is limited. Our aim is to offer insights for the advancement of modern agriculture in ecologically fragile karst tea gardens, as well as microbiomics concepts for green and sustainable environmental development. This study utilized the internal transcribed spacer high-throughput sequencing technology to explore the symbiotic relationship between rhizosphere fungi and plant disease feedback in multiple tea estates across the Guizhou Plateau. The ecological preferences and environmental thresholds of fungi were investigated via environmental variables. Furthermore, a correlation was established between different taxa and individual soil functions. Research has indicated that tea leaf blight disrupts symbiotic connections among fungal groups. For various taxa, we found that numerous taxa consistently maintained core positions within the community, whereas rare taxa were able to stabilize due to a high proportion of positive effects. Additionally, abundant taxa presented a wider range of environmental feedback, whereas the rare taxon diversity presented a stronger positive association with the soil Z score. This study contributes to our understanding of the importance of rare taxa in plant rhizosphere soil processes. Emphasis should be placed on the role of rare taxa in pest and disease control within green agriculture while also strengthening systematic development and biogeographical research related to rare taxa in this region.

**IMPORTANCE:**

In this study, based on internal transcribed spacer high-throughput sequencing, fungal communities in the rhizosphere soil of tea trees and their interactions with the environment in karst areas were reported, and the symbiotic relationships of different fungal taxa and their feedback to the environment were described in detail by using the knowledge of microbial ecology. On this basis, it was found that tea tree diseases affect the symbiotic relationships of fungal taxa. At the same time, we found that rare taxa have stronger cooperative relationships in response to environmental changes and explored their participation in soil processes based on fungal trait sets. This study will provide basic data for the development of modern agriculture in tea gardens and theoretical basis for the sustainable prevention and control of tea tree diseases.

## INTRODUCTION

The population characteristics of ecosystems often include a high concentration of abundant taxa (AT) groups, a large number of rare taxa (RT), and low niche occupancy rates (1), which are widespread from the macro population to the micro world (2, 3). Previous studies have shown that identifying the diverse distribution patterns, relationships, and functional properties of AT and RT in various natural ecosystems (4, 5) and social phenomena (6) is always possible. Therefore, exploring the interaction between AT and RT and their involvement in environmental processes can help researchers understand microbially driven ecological processes and functions.

Microbial communities participate in soil processes and geochemical cycles (7) and are essential for maintaining ecosystem stability (8). With constant advancements in the microbial sequencing technology, studies on the interaction mechanisms between distinct microbial groups and their coupling with the environment have become popular. Recent studies have shown that bacterial diversity and specific bacterial groups are key driving factors for soil multifunctionality in temperate arid and semiarid mountain ecosystems (9). The interaction between pathogenic and mycorrhizal fungi in soil networks can explain the coexistence of aboveground and underground biological communities in forest ecosystems (10). In eukaryotes, fungi are very different from bacteria in prokaryotes, such as filamentous fungi that grow on hyphae and are larger in size (11). Certain types of fungi in the soil infiltrate plant tissues through plant roots or spread at the spore level, forming extensive symbiotic relationships with host plants (12, 13). Moreover, fungi play a fundamental ecological role in mediating plant mineral nutrition and alleviating nutrient limitations in other organisms and constitute a key group in ecological restoration research in ecologically fragile areas. The high physiological and morphological plasticity of fungi can improve the availability of soil nutrients and the absorption of crop nutrients along environmental gradients (14). However, there is limited research on the rich and rare taxa in agricultural ecosystems in ecologically fragile areas, such as karst areas, and little is known about the interaction mechanisms of rare taxa in soil fungal communities and their interactions with abiotic factors.

Tea is the oldest and most popular caffeinated beverage worldwide, with tremendous economic, therapeutic, and cultural significance (15). The Guizhou Plateau has a long history of tea tree (*Camellia sinensis*) cultivation (16, 17), with unique karst landforms, diverse climates, and abundant precipitation, which effectively protect tea tree resources in this region (18). *Colletotrichum camelliae* is a phytopathogenic fungus that causes brown blight in tea trees (19). This disease results in significant production and economic losses to the yield of some sensitive cultivated tea varieties (20). Currently, there are few studies on the rhizosphere fungi of tea gardens in karst areas affected by disease, and it is not clear how different taxa of fungi interact with each other and how they participate in environmental processes. It is understood that disease-induced changes in plant performance can trigger a series of indirect changes in the rhizosphere environment, significantly affecting the composition and assembly mechanism of the rhizosphere microbial community (21). Owing to the low ecological carrying capacity of karst regions (22), the transformation of traditional agriculture to a modern agricultural development model is particularly urgent (23), and green and sustainable agricultural measures need to be further optimized. Therefore, theoretical studies on the diversity of rhizosphere fungi in tea trees and their interactions are particularly important.

This study aimed to examine (i) the composition and diversity of rhizosphere fungal communities under the influence of tea brown blight disease, (ii) the occupancy and co-occurrence relationships of different fungal taxa, (iii) the ecological preferences of abundant and rare taxa groups and their response to the environment, and (iv) the relationships between different fungal taxa and individual soil functions. The research results provide support for the geographical distribution of soil microorganisms in tea gardens in karst areas and for green prevention and control in the future.

## RESULTS

### General distribution patterns of different fungal taxa

**Fig 1:**
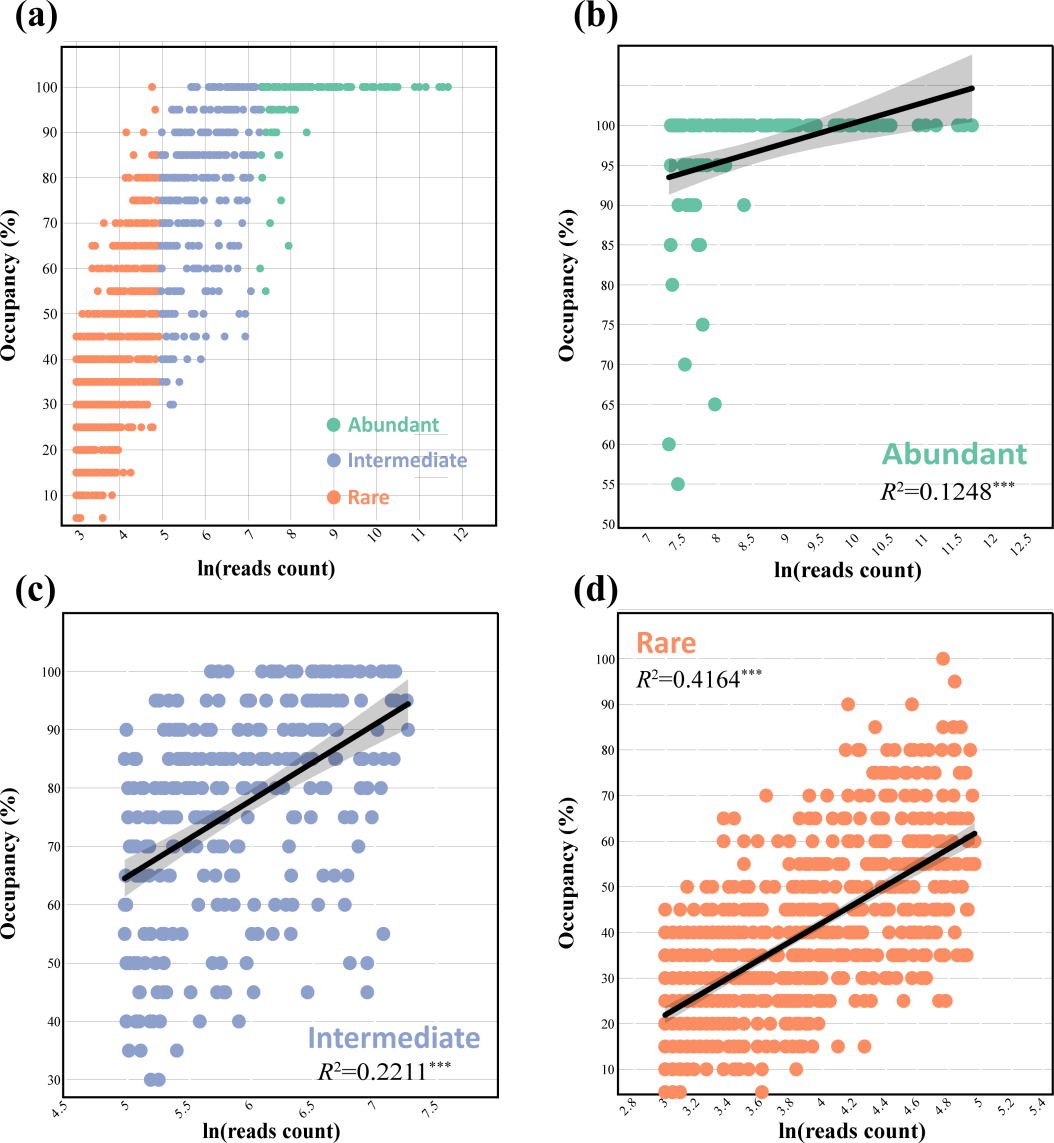
Abundance‒occupancy relationships between abundant taxa and rare taxa from the tea tree rhizosphere. The Spearman correlation coefficient (**R**) was used to quantify the strength and direction of these relationships, with ^***^ indicating statistical significance at *P* < 0.001.

We first described the fungal community and its diversity and evaluated the abundance occupancy relationship of different fungal taxa, as well as the differences in fungal species in the rhizosphere soil between healthy and diseased tea trees. After rigorous data processing (flattening the sequencing data and filtering out OTUs with reads less than 20 to reduce the ground error caused by low-frequency OTUs in subsequent analysis), 1091 OTUs belonging to 14 phyla, 34 classes, 70 orders, 127 families, and 199 genera were identified. At the phylum level, the top three phyla in terms of relative abundance were Ascomycota (51.05%), Basidiomycota (23.69%), and Mortierellomycota (3.99%), with the exception of unclassified OTUs, and the composition of the main fungal phyla was generally consistent within a given species (GSP: given species; GSP includes abundant taxa [AT], intermediate taxa [IT], and rare taxa [RT]) (Fig. S1 to S3). For the GSP, the richness of the categorical ordinals annotated as RT > IT > AT was as expected, and a large portion of the OTUs were classified as RT (62.33%), whereas AT accounted for 10.17% of the total OTUs (Table 1). The abundance occupancy relationship can be used to generate supporting hypotheses for core microbiome members from microbial data sets, prioritizing these taxa for subsequent research. The abundance–occupancy relationship (Fig. 1) indicated that RT had a stronger positive correlation than the other taxa (RT > IT > AT), with all ATs occupying more than 50% of the loci. At 100% occupancy sites, there was only one OTU of RT, whereas AT occupied the vast majority of 100% of the sites. The α diversity data revealed that tea leaf blight affected the fungal community diversity, but this effect was not significant (Fig. S4).

**TABLE 1:** Quantitative statistics of different taxa of tea rhizosphere fungi at various taxonomic levels

Taxa	OTUs	Phylum	Class	Order	Family	Genus
Whole	1091	14	34	70	127	199
AT	111	3	8	20	34	41
IT	300	9	20	43	67	76
RT	680	14	34	62	109	137

Orthogonal partial least squares discriminant analysis (OPLS-DA) is a multivariate statistical analysis method mainly used for classification and feature selection, which can effectively identify the key variables that affect group classification. Using OPLS-DA, we investigated differences in different fungal taxa in the rhizosphere soils of healthy and diseased tea trees. The results indicate that in AT, differential statistical screening identified a downregulated OTU (t test, *P* < 0.05) (Fig. S5a), and the OPLS-DA score better distinguished between healthy and diseased soil samples (R2Y = 0.863, Q2Y = 0.204), explaining 86.3% of the classification information (Fig. S5b). The permutation test revealed that the slopes of the two fitting lines were positive (R2 = 0.79 and Q2 < 0), indicating a certain degree of overfitting (Fig. S6d). Notably, RT screened more OTUs (Fig. S6a), which had a certain degree of overfitting in OPLS-DA (Fig. S6b and d). At the same time, the distance between samples was smaller, but there were more species dispersion values and less aggregation than in AT (Fig. S5c and S6c).

### Rhizosphere fungal ecological co-occurrence network analysis

**Fig 2:**
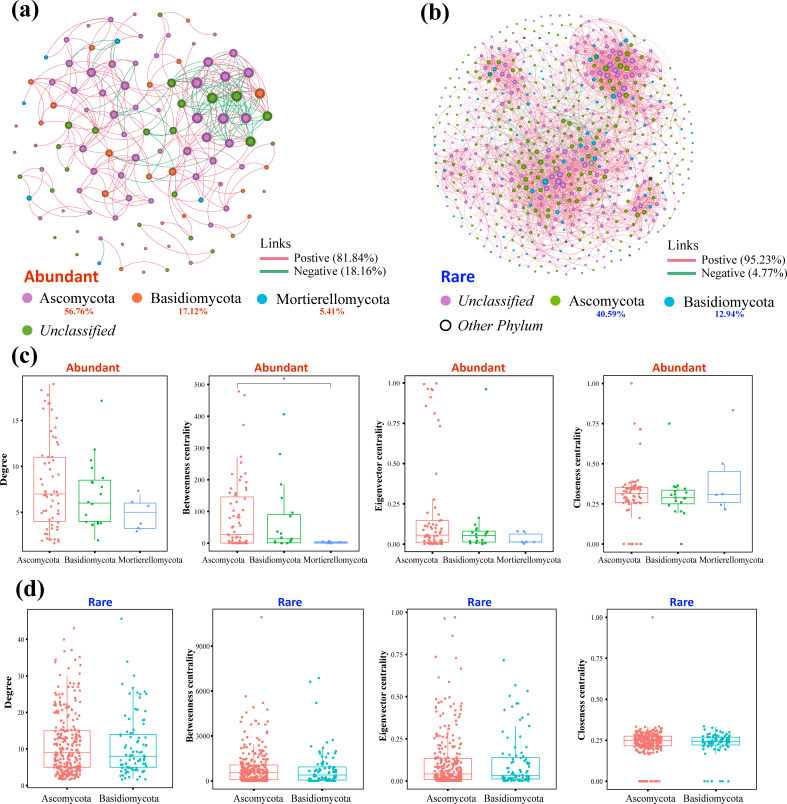
Analysis of the co-occurrence network and the topological structure based on the GSP of tea rhizosphere fungi. (a) Network relationships of AT: each node’s size corresponds to its degree or the number of connections; the color of connections between two nodes represents a positive (red) or a negative correlation (green) displaying the top three ranked phyla; (b) network relationships of AT displaying the top two ranked phyla; (**c and d**) Rich and rare classification group network topology data (degree, betweenness centrality, eigenvector centrality, and closeness centrality) box plots, Wilcoxon test for difference analysis, * means *P* < 0.05.

Cooccurrence networks were used to analyze the potential interaction relationships of the GSP. AT had more nodes and links than RT (Table S1). The average weighted degree, graph density, and average clustering coefficient of AT were greater than those of RT (Table S2; Fig. 2). Notably, the species in RT presented a more positive (95.23%) tendency toward cooperation. Compared with AT, RT results in a larger and more complex network topology with an increase in the number of links and nodes (Fig. 2b). From the perspective of the topological structure at the phylum level, the AT network exhibited ladder-like characteristics, where dominant species dominated (Fig. 2c). Although Ascomycota accounted for 40.59% of RT, there was no dominant tendency, and its topological center parameters were not significantly different from those of Basidiomycota (Fig. 2d).

**Fig 3:**
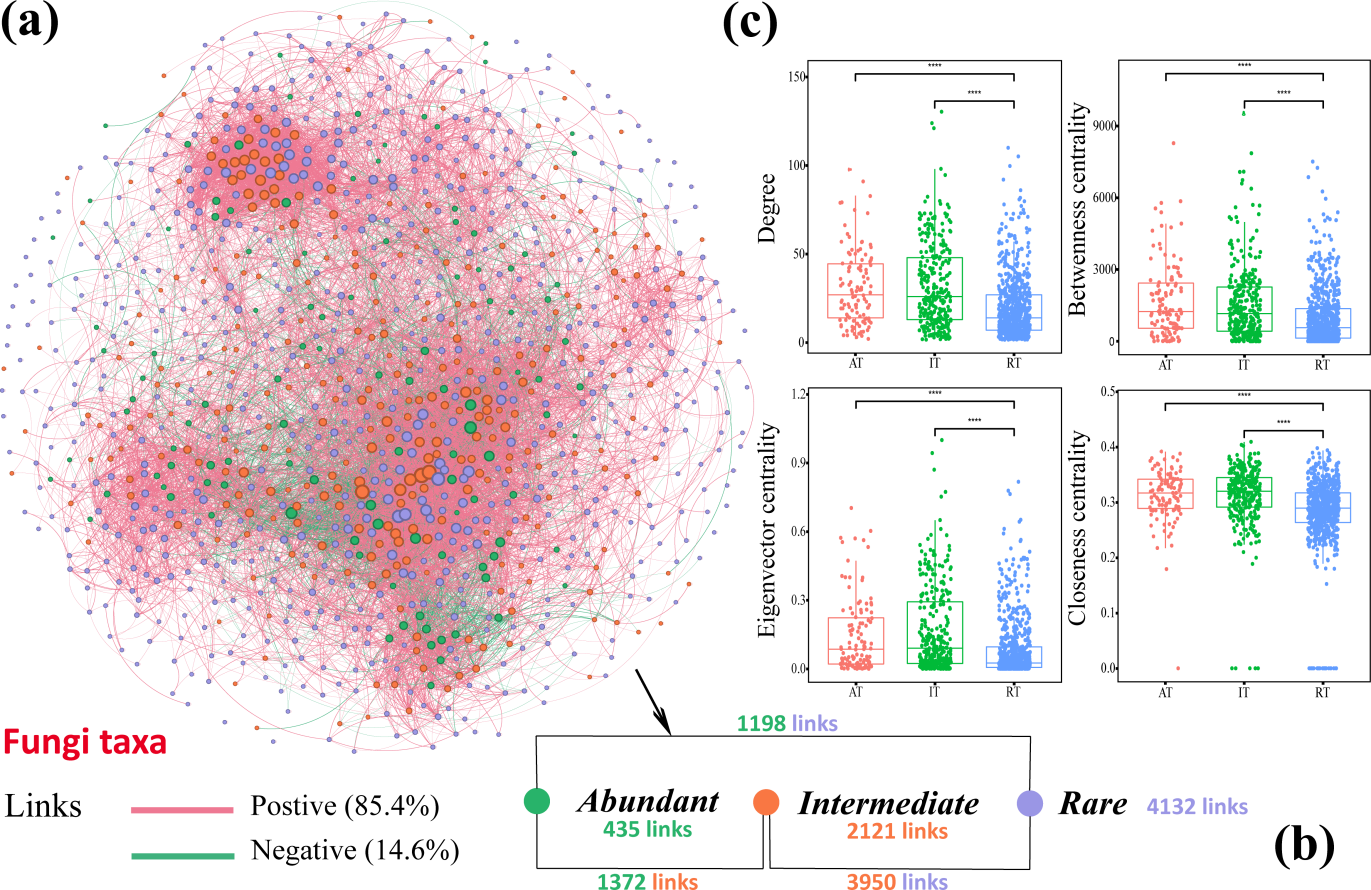
Analysis of the co-occurrence networks and topological data differences for GSP. (a) Network analysis reveals relationships both within and across GSPs. A link represents a significant (FDR-corrected *P* < 0.05) and strong (Spearman’s |r| > 0.65) connection; (b) the number of links within and between GSPs; and (c) analysis of differences in the network topology indicators between GSPs using the Wilcox test for significance testing. *** indicates *P* < 0.001.

The fungal co-occurrence network can reveal the ecological relationships between fungal communities and speculate on the interactions between species. Here, we constructed a fungal network using correlation coefficients |r| > 0.65 and *P* < 0.05 to characterize the interaction relationships between GSPs (Table S2; Fig. 3a). In fungal communities, there are extensive positive correlations (positive) between different taxa (AT, IT, and RT), with a high proportion of 95.23% between IT and RT, but the proportion of positive correlations between RT and AT is the smallest (Table 2; Fig. 3b). Notably, although RT has a large group, its cooperative relationship with AT is weaker than that between IT and AT (links: 1198 < 1372), whereas there is a broad connection between IT and RT (links: 3950). From the perspective of topology, the position of AT as the dominant species was not reflected in the topology data, and its difference from that of IT was not significant. However, the topology data for RT were significantly related to both AT and IT (Fig. 3c). Under the influence of tea leaf blight, there was a downwards trend in the positive correlation links in the fungal network, and the main topological data were lower than those in the healthy sample network (Table S3).

**TABLE 2:** Statistical analysis of the correlation between different taxa (GSP) of tea rhizosphere fungi

GSP	Total links	Positive links	Negative links	Positive correlations (%)
AT-AT	435	356	79	81.84
AT-IT	1372	1073	299	78.21
AT-RT	1198	919	279	76.71
IT-IT	2121	1741	380	82.08
IT-RT	3950	3255	695	82.41
RT-RT	4132	3935	197	95.23

### Response of different fungal taxa to the environment

**Fig 4:**
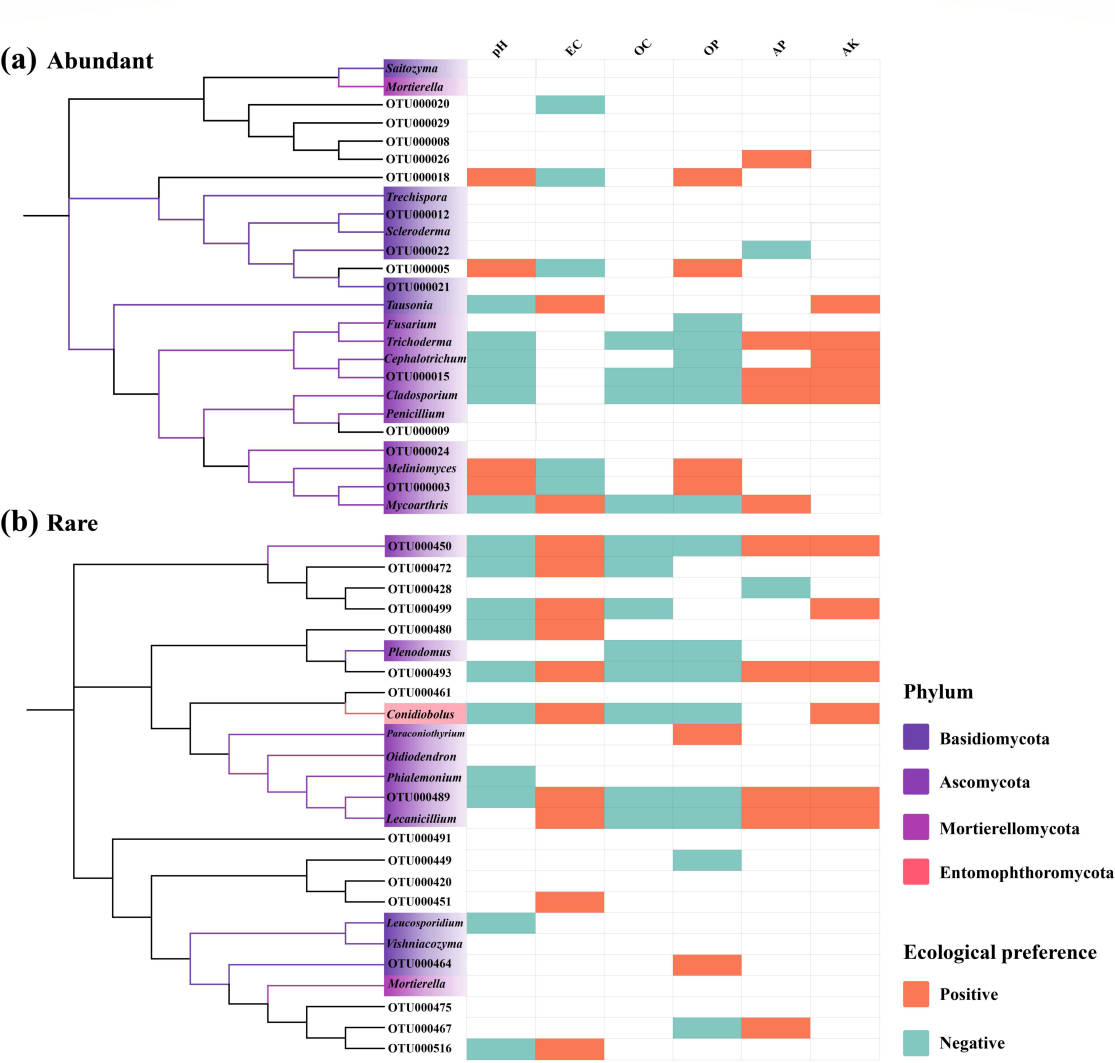
Correlations between GSPs (AT and RT) and the environment of fungi in the rhizosphere soil of tea trees. (**a and b**) Phylogenetic relationships and environmental preferences of the top 25 OTUs in terms of the relative abundance in AT and RT. The neighbor-joining method was used to construct the phylogenetic tree. Taxa that are genus-level assignable are shown; those that are not are displayed as OTU IDs.

In order to clarify the relationship between soil fungi and the environment, we not only measured the hydrogen (pH), electrical conductance (EC), and soil organic matter (SOM) that are closely related to soil quality, but also emphasized the mutual relationship between soil available nutrients (quick-acting phosphorus, available potassium, and alkaline hydrolyzed nitrogen) and fungal communities. To obtain information on potential traits, we attempted to define the ecological preferences of each OTU via Spearman’s correlations between fungal taxa and environmental variables (Table 3). Fungal community traits were acquired via the “Hmisc” package in R. Here (Fig. 4), both AT and RT presented similar phylogenetic diversities, although they were dominated by Basidiomycota, Ascomycota, and Mortierellomycota. Among them, AT included 12 genera of fungi, including *Saitozyma*, *Fusarium*, and *Trichoderma*, whereas the RT group included nine genera of fungi. Moreover, the correlation between rare taxonomic groups and the environment was slightly greater than that between AT (ecological preference point RT: 50 > AT: 42) and the environment. Notably, both AT and RT had identical ecological preferences for OC (negative) and AK (positive) in terms of their OTU traits (Fig. 4). In RT, except for OTUs that did not display ecological preferences, the ecological preferences of the other OTUs for pH (native) and EC (positive) were completely consistent (Fig. 4b).

**TABLE 3:** Ecological preferences between different taxa of fungi and environmental variables (fungal community traits)^*a*^

Tax/Env		pH			EC			OC			OP			AP			AK		
		+	NA	−	+	NA	−	+	NA	−	+	NA	−	+	NA	−	+	NA	−
AT	OTUs	16	72	23	25	71	15	5	83	23	21	71	19	19	90	2	19	88	4
	Average	−0.0631			0.0901			−0.1622			0.0180			0.1532			0.1351		
IT	OTUs	46	167	87	85	172	43	20	215	65	37	206	57	43	252	5	61	219	20
	Average	−0.1367			0.14			−0.15			−0.6667			0.1267			0.1367		
RT	OTUs	69	487	124	121	513	46	42	550	88	43	545	92	48	614	18	78	565	37
	Average	−0.0809			0.1103			−0.0676			−0.0721			0.0441			0.0603		
Total	OTUs	131	/	234	231	/	104	67	/	176	101	/	168	110	/	25	158	/	61

^*a*^ For example, the positive and negative correlations of OUT with pH were defined as acid- or alkaline-preferred correlations. “+” is denoted as “1” in the data set representing a positive correlation; “NA“ is denoted as “0“ in the data set representing no correlation; and “−” is denoted as "−1" in the data set representing a negative correlation; “/” means that the indicator is not applicable here.

**Fig 5:**
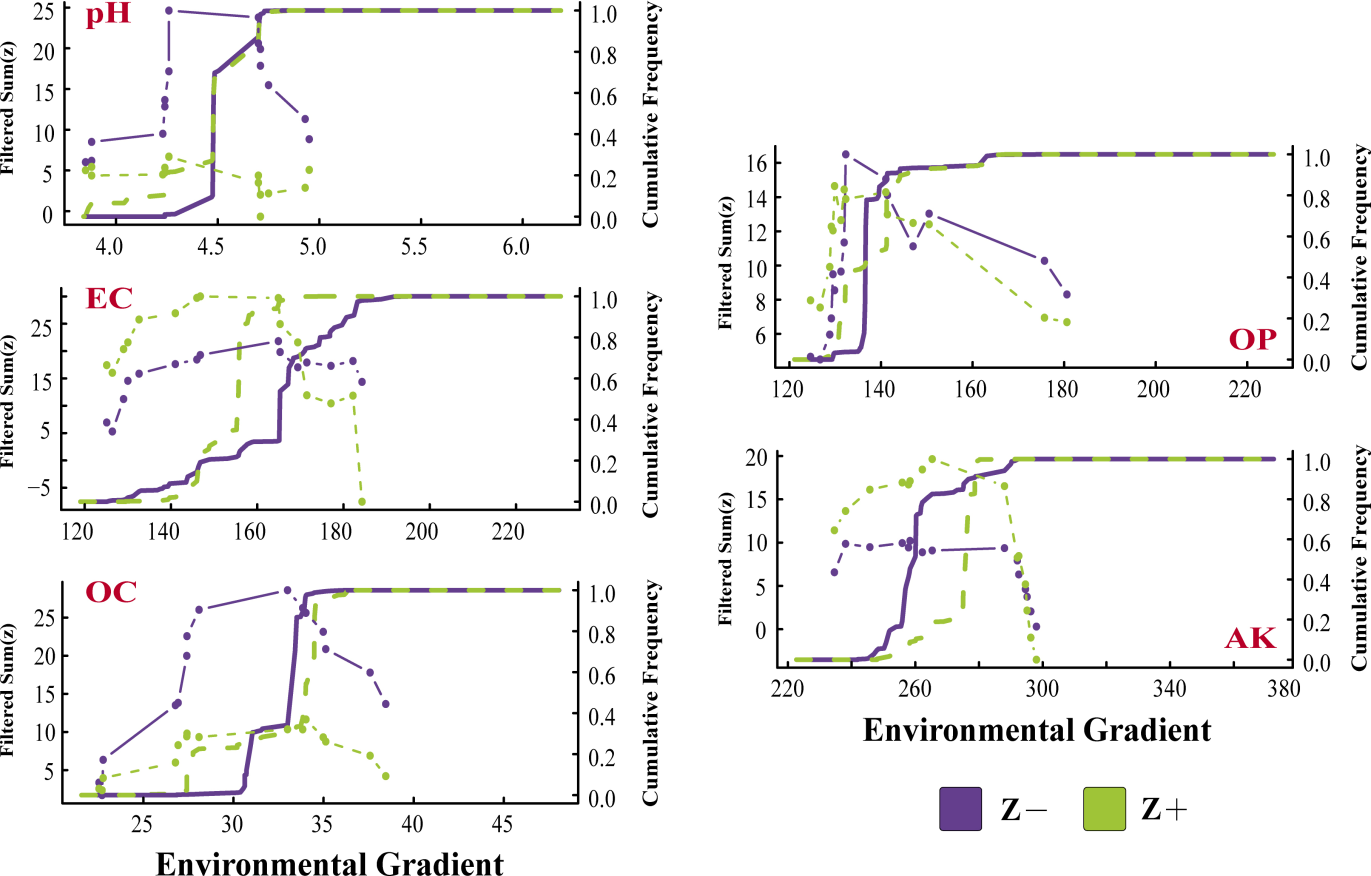
Response curves of the fungal AT-negative responsive species (Z−) and -positive responsive species (Z+), indicating the total scores along environmental gradient mutation points. All community members' z scores are displayed. The green symbols represent the rising taxa (Z+), whereas the violet symbols represent the declining taxa with increasing environmental gradient (Z−).

**Fig 6:**
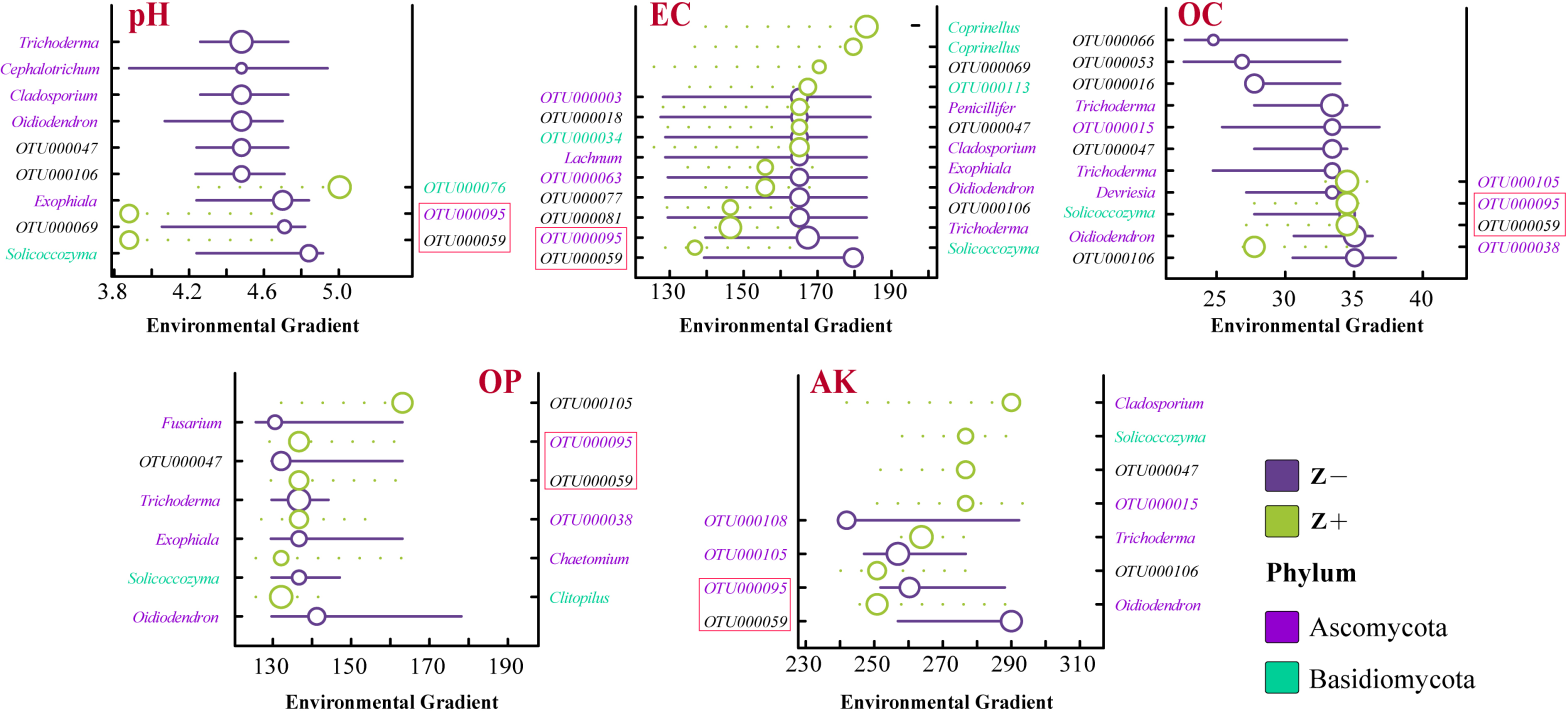
Distribution of the negatively and positively responsive species in communities along environmental gradients. OTUs that have been identified as genera are represented by their genus names; otherwise, the OTU ID is displayed.

To identify the environmental thresholds for fungal communities in relation to each variable, we assessed the accumulating Z+ (positive response species generated by changes along environmental gradients) and Z− (negative response species generated by changes along environmental gradients) points of change via the “TITAN2” package (Fig. 5 and 6). We did not add IT and RT here to eliminate the interference caused by the “double zero problem.” The results revealed that the response of reducing taxa to EC enrichment lagged behind that of increasing taxa, whereas the process of AK enrichment had the opposite effect. The overall responses of most species to the various environmental gradients were consistent (Fig. 5). Moreover, the distribution maps of negatively and positively responsive species along the environmental gradient revealed that the gradient changes in pH and OC caused an increase in negatively responsive species, whereas EC and AK showed opposite trends (Fig. 6). Notably, all the response species, with the exception of the unidentified OTUs, belonged to Ascomycota and Basidiomycota, and *OTU000059* (unclassified) and *OTU000095* (Ascomycota) responded to all the environmental variables (Table S4; Fig. 6).

**Fig 7:**
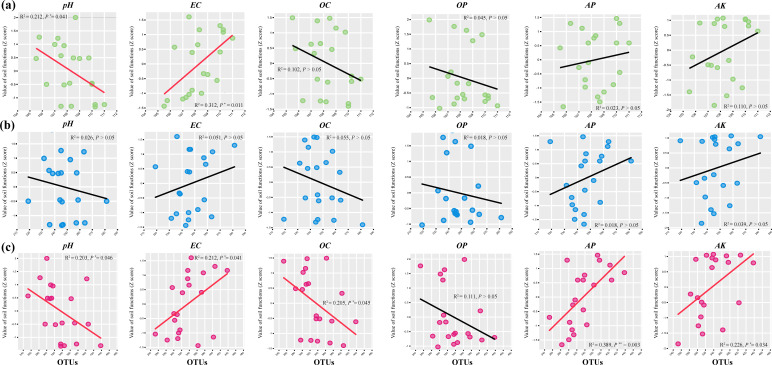
Relationships between AT (a), IT (b), and RT (c) α diversity (*Chao 1* index) and individual soil function (*N* = 20). The lines represent the fitted linear ordinary least squares (OLS) model. The red and black lines denote statistically significant (*P* < 0.05) and nonsignificant (*P* > 0.05) relationships, respectively. Note: “**” means *P* < 0.01; “*” means *P* < 0.05.

**Fig 8:**
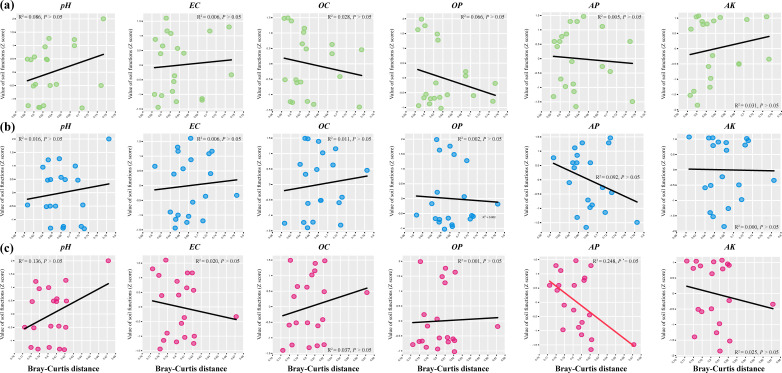
Relationships between AT (a), IT (b), and RT (c) β diversity (Bray‒Curtis distance) and individual soil function (*N* = 20). The lines represent the fitted linear ordinary least squares model. The red and black lines denote statistically significant (*P* < 0.05) and nonsignificant (*P* > 0.05) relationships, respectively. Note: “*” indicates *P* < 0.05.

We further evaluated the relationships between GSP diversity (α and β diversities) and individual soil functions (Table S6; Fig. 7 and 8). By evaluating the relationships between α diversity and individual soil functions through an overall sample analysis (*N* = 20), we found a significant negative correlation between soil pH and both AT (*R*^2^ = 0.212, *P* < 0.05) and RT (*R*^2^ = 0.203, *P* < 0.05). Soil EC, an important indicator, was significantly positively correlated with both AT (R^2^ = 0.312, *P* < 0.05) and RT (*R*^2^ = 0.212, *P* < 0.05). Interestingly, the RT α diversity was also significantly positively correlated with soil AP (*R*^2^ = 0.389, *P* < 0.01) and AK (*R*^2^ = 0.226, *P* < 0.05) and significantly negatively correlated with soil OC (*R*^2^ = 0.205, *P* < 0.05). However, there was no significant correlation between the IT (*P* > 0.05) and individual soil functions. From the β diversity perspective, only RT had a significant negative correlation with soil AP (*R*^2^ = 0.389, *P* < 0.05), whereas the other GSPs were not significantly correlated with individual soil function.

## DISCUSSION

### Tea tree diseases lead to the emergence of indicator species in different taxa of rhizosphere fungi

Soil is an essential biological matrix in nature, and plant root exudates that are released into the soil can affect soil properties. Rhizosphere and ectorhizosphere soils were the first to respond to this phenomenon because it was proved that root exudates from plants can enhance the abundance of beneficial taxa for specific plant species, thereby affecting the composition and function of soil microorganisms (24). This study revealed that the available nutrient content was affected by tea tree diseases. The AP and AK contents of healthy samples were slightly lower than those of diseased samples (Table S1) possibly because tea tree diseases cause poor absorption of nutrients that cannot be utilized. Moreover, certain pathogenic microorganisms can also consume nutrients in the soil, leading to nutrient imbalance. This phenomenon has also been observed in previous studies (25). Importantly, unhealthy tea tree soil frequently becomes acidified (Table S1), which directly changes the soil environment and leads to changes in the beneficial microbial population (26). These changes are not conducive to the reproduction of beneficial microorganisms but accelerate the emergence of other harmful fungi (27, 28). This indicates the importance of management methods based on modern agriculture as a basic concept by utilizing ecological principles and methods. Consequently, the stability of ecosystems and the sustainability of agricultural production can be achieved. Organic fertilizers, pesticides, and biological control methods can be used to improve the soil quality, protect the ecological environment, promote biodiversity, and enhance the quality of agricultural products, further reflecting the irreplaceability of soil as a biological substrate.

Ascomycota and Basidiomycota were the main phyla of fungi belonging to GSP (Fig. S1 to S3), indicating that fungal taxa have a certain degree of stability at relatively high levels and are not affected by tea tree diseases. In GSP, compared with that in IT, the proportion of Ascomycota in AT generally decreased, whereas that of Basidiomycota generally increased. Some members of the phylum Basidiomycota form mycorrhizal fungi in symbiosis with plants (29), which is beneficial for crop cultivation. Some basidiomycetes can cause diseases in forests and garden plants (30), resulting in economic losses. The abundance‒occupancy relationship is a critical indicator for studying fungal community relationships as a core or host-specific group. A greater abundance of AT had a wider distribution, whereas a lower occupancy rate of RT implied a greater elimination risk (Fig. 1). Previous studies support this viewpoint (31). In addition, OPLS-DA demonstrated (Fig. S5 to S6) that tea tree diseases have a significant effect on fungal communities, and AT screening revealed that the genus *Penicillium* was significantly present in the affected samples (M). *Penicillium* belongs to the heterotrophic aerobic type, which can cause plant *Penicillium* disease, causing the formation of large areas of disease spots in plants and plant death in severe cases (32). In RT, two families, namely, Bulleribasidiaceae and Phyllosticaceae, were present in the diseased sample (M), and Phyllosticaceae can cause plant leaf blight (33).

### Compared with nonfungi, fungi have relatively high symbiotic rates

Microbial network relationships are driven by multiple factors, such as crossfeeding, legacy effects, and environmental filtering (34). Microorganisms often exist in symbiotic forms that are conducive to the construction of complex ecological networks (35). Our results revealed a relatively positive correlation in the RT (95.23%) network, and the significance test of the topological structure revealed that AT still played a core role in the community (Table S2; Fig. 2). Previous studies on the network relationships of soil microbial communities in the Hexi Corridor region of China have shown that most of the network links within the taxa are positively correlated (36), which is consistent with our findings.

RT is crucial for the construction of microbial networks and serves as an indicator of the evolution of soil processes and vegetation succession (4, 5). In this study, we found that the internal connections between the RT taxa were the closest and mostly positive (Fig. 3a and b). Studies have shown that rare taxa have important ecological functions, including element cycling, pollutant degradation, and host health (37). There were fewer connections between AT and RT, and the proportion of negative correlations was greater than that between the other groups (Table 2). RT was located at the center of the network and tended to cooperate with the intermediate taxa (IT) (Fig. 3a and b). The reasons for their lower cooperation with AT are partly their abundance‒occupancy relationships (Fig. 1) and partly the key role that they may be playing in enhancing the stress resistance of fungal communities, as well as maintaining their structure and stability (38). The key role of RT is self-evident. Generally speaking, abundant and rare taxa exhibit different responses to environmental changes, while rare taxa are more sensitive (39). Studies have shown that RT with flexible and diverse taxa can improve the selection efficiency of key taxa rather than relying solely on the input of new microbial taxa under environmental interference (40). Notably, tea leaf blight increased the negative correlation between species of different taxa in the fungal network, and the topological structure became loose (Table S3). This means that the resource competition between taxa caused by plant diseases intensifies, thereby disrupting community stability.

### Environmental response of abundant and rare taxa

Soil microbes have significant ecological functions in the soil nutrient cycle and plant mineral nutrition (41). However, because of their susceptibility to environmental changes, external environmental changes frequently result in changes in variety and community structure (42, 43). Here, we discuss the ecological preferences of soil microorganisms for the external environment. First, we observed that, compared with RT (19.75%) (Table S5), AT (28.68%) exhibited a broader ecological preference for environmental variables. This can also explain why, compared with RT, AT has greater adaptability to environmental changes and can effectively utilize a wider range of resources. Research on other agricultural ecosystems supports this view (31). Interestingly, among the top 25 OTUs in terms of the relative abundances of AT and RT, the response of RT to the environmental variables was slightly greater than that of AT (Fig. 4). This phenomenon can be explained by the efficient selection of RT, which can stabilize it in the community and increase its occupancy rate, rather than by continuously updating RT through environmental intervention. In addition, the response traits of the AP and AK preferences in fungal communities are relatively conserved (Table S5; Fig. 4), and previous studies have shown that root exudates can affect soil available nutrients during plant growth stages (44). Therefore, revealing the ecological preferences of fungal communities for available nutrients can be used to evaluate the effects of soil feedback on plant growth. Soil microorganisms affect the transformation and supply of nitrogen and other nutrients. An environmental threshold analysis revealed more negatively responsive species along the pH gradient in AT compared with positively responsive species (Fig. 5 and 6). This is due to the fact that tea trees are well-suited for acidic soil, and environments with excessively high or low pH levels hinder nutrient absorption (45, 46). A reasonable pH (4.0–4.8) is beneficial for improving disease resistance.

This study focuses on the relationships between GSP and the individual soil functions of different environmental variables (Fig. 7 and 8). Standardized Z-scores for the environmental parameters revealed that individual soil functions and fungal α diversity differed surprisingly across taxa, as supported by similar studies on bacteria and fungi (9, 47). There are several possible explanations for this phenomenon. First, the linear relationship between fungal community diversity and individual soil functions depends on the proportion of positive and negative species that respond to environmental gradients. Our ordinary least squares fitting trend was consistent with the environmental threshold analysis, which explains this issue. Second, as the relative abundance and occupancy of species increase, certain species with specific functions in relatively high-abundance taxa (AT and IT) may play a reduced role in the community, and the active cooperation of RT gradually increases the community stability. Another explanation is that AT and plant roots compete for scarce nutrients, especially in karst areas where nitrogen and phosphorus limitations were more prominent in our study. This hypothesis is supported by previous research (48). In addition, this phenomenon indicates that the relationships between different fungal taxa and individual soil functions manifest as differences in the ecological niche complementarity and stochastic processes of community ecology (49).

From the perspectives of fungal involvement in co-occurring network construction and soil ecological functions, the interaction between rare fungal groups is crucial for determining the community composition and maintaining the ecosystem multifunctionality (50). Our research has found that species within rare taxa have more positive interactions, indicating the importance of species interactions within rare fungal subpopulations in supporting ecosystem function and stability (51). In addition, the cooperation of rare taxa may play a crucial role in their survival in tea garden soil (acidic). Most fungi characterized by hyphal growth are interconnected and form a network through hyphae, providing timely feedback on environmental changes, which helps share resources and coordinate microbial activities (52). Therefore, closely monitoring the cooperation between rare fungal groups can provide solutions for environmental disturbances, including plant diseases and extreme weather events, and enhance the resilience of microbial communities, even soil quality (53).

### Conclusions

Our study mainly focused on disease-mediated fungal changes in the rhizosphere soil of tea trees in karst tea garden ecosystems, as well as the interaction mechanisms of different fungal taxa and their responses to the environment. Our results provide reliable evidence that tea tree diseases increase fungal species richness but decrease fungal diversity, and that the distance between samples is significantly different. The results also revealed that the abundance of AT is closely related to its core position in the community, while the positive relationship between RT enables it to be stable in the community rather than being input from the external environment, which helps us focus on RT in maintaining plant root health. Importantly, RT has a more linear relationship with environmental variables, which also means that it plays a positive role in soil processes and their interactions with plants, especially in karst areas where carbon and nitrogen limitations are more prominent. In this study, we particularly emphasized the symbiotic relationship between RT and its response to the environment. The study of rhizosphere soil in tea gardens in karst areas via a large-scale geographic analysis will contribute to understanding the biogeographic pattern of RT and its relationship with the environment as a reference for the refined management of modern agriculture in karst areas.

## MATERIALS AND METHODS

### Study sites

This study was conducted in a tea garden (26°51′75″N, 106°38′38″E, and 27°08′47″N, 107°35′16″E) in the Qianzhong Karst Plateau area in 2023 during a period of high incidence of tea tree diseases (June). The study area is located in the highland area of the dissolved mound depression, with typical subtropical humid tea garden ecosystems. Four sampling sites were set up in Qingzhen City (QS) and Weng’an County (WS), covering both the healthy (H) and diseased (M) areas of the tea gardens, with five biological replicates set up at each site and basically the same cultivation history and species in all the tea gardens (Fig. S7).

### Sample collection

All four sampling sites were located in areas with a relatively rich vegetation diversity. Sampling sites H and S were selected according to the actual tea tree incidence. The sampling sites, where the vegetation communities were relatively well established, were chosen to minimize the impact of human activities and animal infestation. Four sampling points (four points × each point sampled via the five-point method) were chosen. Each biological replicate consisted of a mixture of rhizosphere soils from a single mini-sample plot (1 m × 1 m), with a distance of at least 50 m between each sampling plot.

After the samples were prepared according to the sample plot and sample point setup, we collected rhizosphere and ectorhizosphere (for the determination of soil environmental factors) soil samples from healthy and diseased tea trees for subsequent analyses on the basis of microbial sequencing via internal transcribed spacer (ITS) sequencing and determination of soil chemical properties, respectively. The disinfection of the shovels and medical scissors with 75% alcohol and of the sampling equipment and the replacement of medical gloves were required for each sampling session. The rhizosphere soil samples used for field collection were as follows: (1) the root system of the tea tree was cut; the attachments were shaken in time; and 1 mm of attached soil was retained; and (2) 50 mL centrifuge tubes were filled, and the samples were stored in a liquid nitrogen tank to bring them back to the laboratory for spare parts. Ectorhizosphere soil collection: after the tea tree roots were obtained, excess soil was manually shaken from the roots and thoroughly homogenized to form a mixed sample representing the ectorhizosphere soil (a 2 mm-diameter mesh sieve was used to filter the impurities, gravel, and apomictic material from the ectorhizosphere soil). Rhizosphere soil was extracted from field-collected roots in the laboratory. The roots were placed in a 50 mL centrifuge tube and washed with 3 mL of phosphate-buffered saline at 180 rpm for 20 min to remove the soil from the root surface. The mixture was subsequently centrifuged at 4000 rpm for 20 min; the supernatant was removed; and the sediment was collected for extraction. The ectorhizosphere soils were dried naturally, and then chemically characterized.

### Determination of soil chemical properties and functional assessment

The soil organic matter (SOM) content was assessed using an external heating method with potassium dichromate, while the alkali diffusion method was employed for determining the soil alkali-hydrolyzable nitrogen (AN). For the assessment of soil available phosphorus (AP), sodium bicarbonate leaching combined with molybdenum–antimony colorimetry was utilized, and ammonium acetate leaching, followed by atomic absorption spectrometry, was applied to detect soil available potassium (AK). The pH and the electrical conductivity (EC) of a soil–water slurry at a 1:2.5 weight-to-volume ratio were measured according to established protocols (54). The results are presented in Table S7. In this study, six soil indicators (pH, EC, SOM, AN, AP, and AK), which are good indicators of tea tree soil productivity, fertility, and other factors in tea gardens, were used to assess individual soil functions. The relationships between biodiversity and soil functioning have been characterized in previous studies via single functions (55), turnover (56, 57), averaging (58), and single thresholds (59). Individual soil functions were quantified via Z-score conversion (9).

### Meta-second-generation amplicon

#### DNA extraction and PCR amplification

DNA was extracted from the target soil samples via HiPure Soil DNA Kit (Magen, Guangzhou, China) according to the manufacturer’s instructions. The fungal ITS1 region was amplified via the primer pair ITS1 F KYO2 (5′-TAGAGGAAGTAAAAGTCGTAA-3′)/ITS86R (5′-TTCAAAGATTCGATGATTCAC-3′) (60). The related PCR reagents were purchased from New England Biolabs (USA).

#### Illumina sequencing

The amplification product quality was assessed via 2% agarose gel, and the PCR products were purified via AMPure XP Beads (Beckman, CA, USA) and quantified via Qubit 3.0. The sequencing libraries were constructed via Illumina DNA Prep Kit (Illumina, CA, USA). The library quality was checked via ABI StepOnePlus Real-time PCR System (Life Technologies, Foster City, CA, USA). The qualified libraries were pooled via PE250 mode of NovaSeq 6000 for online sequencing. The raw sequences were submitted to the NCBI Sequence Read Archive (accession number PRJNA1036117).

#### Bioinformatics analysis

FASTP (version 0.18.0) for filtering raw data (61) and FLASH (version 1.2.11) for sequence splicing (clean reads [62] were merged into tags at a minimum overlap of 10 bp and a maximum mismatch rate of 2% threshold), tag filtering with reference to Qiime’s (63) tag quality control processes (64), and UPARSE (version 9.2.64) for clustering dechimeras (65).

### Data processing

OTUs containing fewer than 20 reads were discarded to avoid random effects in RT identification (30, 35). All samples were rigorously standardized prior to the analysis. On the basis of previous studies (66), we defined taxa on the basis of the relative abundance of fungal OTUs: i) OTUs with a relative abundance of more than 0.1% of the total number of sequences were defined as “abundant” taxa (AT); ii) OTUs with relative abundances below 0.01% were defined as “rare” taxa (RT); and iii) OTUs with a relative abundance between 0.01% and 0.1% were considered “intermediate” taxa (IT).

The abundance–occupancy relationship was used to explain the diversity changes and the spatial distributions of different taxa, where the occupancy calculation formula is as follows (67):


$$Occupancy=\frac{{Nsites}_{S,H}}{{Nsites}_{H}}$$

where *S* is the OTU; *H* is the habitat; and occupancy represents the ratio of the number of samples with *S* (Nsites*_S, H_*) appearing in the OTU (*S*) in habitat *H* to the total number of samples with *H* (Nsites*_H_*).

Orthogonal partial least squares-discriminant analysis (OPLS-DA) was used to perform a differential analysis on samples via the “ropls” package (68). A permutation test was performed for external verification. The “reshape2” package was used for the cooccurring network analysis of fungal taxa (use = “pairwise,” method = “Spearman,” adjust = “FDR,” alpha = 0.05; correlation threshold: *r* = 0.65, *P* = 0.05). MEGA 11 was used to process the OTU sequences and construct the phylogenetic treesconstructed via the neighbor-joining method (time = 1000). To obtain potential traits, Spearman from the “Hmisc” package was used to calculate the correlation between the relative abundance of rare or abundant OTUs and environmental variables, determine the ecological preferences of each OTU (69), and form a fungal community trait data set. A chiplot (https://www.chiplot.online/) was used to construct a heatmap of the OTU ecological preferences and phylogenetic trees. The ecological threshold of a community under specific environmental gradients is quantified (minSplt = 3) via the “TITAN2” package (70). The physical and chemical properties of the soil were used to calculate the individual soil functions, and a scale function was used for data standardization. The Z-score calculation formula for the ecosystem parameters (single indicators) is as follows:


$$Z_{ij}=(x_{ij}-\lambda_{j})/\delta_{j}$$

where $Z_{ij}$ is the Z-score of the ecosystem (single indicator) parameter *j* in plot ***i***, with a range of ***i*** between 1 and 20 and a range of ***j*** between 1 and 6; $x_{ij}$ is the numerical value of the ecosystem (single indicator) parameters; $\lambda_{j}$ is the average value of the *j*-th ecosystem (single indicator) parameter at 20 sampling points; and $\delta_{j}$ is the standard deviation of the average value of the *j*-th ecosystem (single indicator) parameter at 20 sampling points.

## Data Availability

The raw sequences were submitted to the NCBI Sequence Read Archive (accession number PRJNA1036117).

## SUPPLEMENTAL MATERIAL

Supplemental material spectrum.01889-24-s0001.docx Tables S1 to S7; Fig. S1 to S7.

ASM does not own the copyrights to Supplemental Material that may be linked to, or accessed through, an article. The authors have granted ASM a non-exclusive, world-wide license to publish the Supplemental Material files. Please contact the corresponding author directly for reuse.
