# Supplementary material for: ITS amplicon sequencing revealed that rare taxa of tea rhizosphere fungi are closely related to the environment and provide feedback on tea tree diseases
Source: Microbiol Spectr. 2024 Nov 29;13(1):e01889-24. doi: 10.1128/spectrum.01889-24 (PMC11705919; doi:10.1128/spectrum.01889-24)

**Table S1** Physical and chemical properties of rhizosphere soil of tea trees

| **Sample** | **pH** | **EC** | **OC** | **OP** | **AP** | **AK** |
| --- | --- | --- | --- | --- | --- | --- |
|  |  | µs/cm | g/kg | mg/kg | mg/kg | mg/kg |
| QS-H1 | 5.43 | 146.8 | 33.84 | 141.05 | 12.2 | 262.21 |
| QS-H2 | 5.06 | 126.4 | 33.00 | 150.50 | 15.2 | 257.87 |
| QS-H3 | 4.95 | 125.1 | 34.98 | 147.00 | 13.6 | 258.35 |
| QS-H4 | 5.08 | 123.8 | 34.02 | 129.85 | 12.0 | 265.31 |
| QS-H5 | 4.93 | 132.6 | 35.12 | 141.40 | 13.8 | 255.9 |
| QS-M1 | 4.70 | 146.0 | 38.45 | 175.70 | 12.6 | 245.73 |
| QS-M2 | 4.71 | 141.0 | 37.58 | 180.60 | 12.9 | 238.11 |
| QS-M3 | 4.71 | 130.0 | 40.58 | 184.10 | 12.4 | 231.12 |
| QS-M4 | 4.75 | 129.0 | 40.03 | 187.25 | 13.1 | 222.67 |
| QS-M5 | 4.7 | 119.0 | 40.68 | 192.50 | 14.4 | 234.6 |
| WS-H1 | 4.26 | 165.3 | 22.14 | 131.95 | 15.6 | 298.03 |
| WS-H2 | 4.26 | 171.5 | 22.54 | 132.30 | 14.8 | 299.37 |
| WS-H3 | 4.23 | 164.9 | 22.71 | 129.50 | 14.6 | 298.55 |
| WS-H4 | 4.24 | 187.8 | 21.57 | 126.70 | 14.6 | 298.69 |
| WS-H5 | 4.24 | 169.4 | 22.77 | 121.10 | 15.4 | 295.10 |
| WS-M1 | 3.88 | 182.2 | 26.93 | 129.15 | 15.4 | 292.49 |
| WS-M2 | 3.85 | 184.3 | 26.78 | 128.80 | 14.9 | 292.04 |
| WS-M3 | 3.85 | 195.3 | 27.41 | 124.60 | 12.8 | 294.58 |
| WS-M4 | 3.84 | 184.3 | 28.09 | 131.25 | 13.4 | 296.26 |
| WS-M5 | 3.88 | 177.1 | 27.42 | 123.20 | 14.6 | 288.00 |

**Table S2** Co-occurrence network topology of GSP

| Tax |  | Node | Links | Average weighted degree | Network diameter | Graph density | Modularity | Average clustering coefficient | Average path length |
| --- | --- | --- | --- | --- | --- | --- | --- | --- | --- |
| AT |  | 111 | 759 | 13.673 | 8 | 0.071 | 0.618 | 0.703 | 3.341 |
| RT |  | 680 | 5508 | 16.2 | 10 | 0.018 | 0.649 | 0.551 | 4.065 |
| Whole |  | 1091 | 19681 | 36.079 | 9 | 0.022 | 0.562 | 0.5 | 3.379 |

**Table S3** Mutual connectivity of co-occurrence networks based on given species (AT, IT and RT).

| **GSP** | **Total links** | **Positive links** | **Negative links** | **Positive correlations (%)** |
| --- | --- | --- | --- | --- |
| AT-AT | 435 | 356 | 79 | 81.84 |
| AT-IT | 1372 | 1073 | 299 | 78.21 |
| AT-RT | 1198 | 919 | 279 | 76.71 |
| IT-IT | 2121 | 1741 | 380 | 82.08 |
| IT-RT | 3950 | 3255 | 695 | 82.41 |
| RT-RT | 4132 | 3935 | 197 | 95.23 |

**Table S4** Co-occurrence network topology of H and M

| Sample | Positive correlations (%) | Average weighted degree | Network diameter | Graph density | Modularity | Average clustering coefficient | Average path length |
| --- | --- | --- | --- | --- | --- | --- | --- |
| H | 70.06% | 73.369 | 4 | 0.066 | 0.42 | 0.442 | 2.413 |
| M | 64.19% | 50.382 | 11 | 0.092 | 0.298 | 0.261 | 3.483 |

**Table S5** Base sequence information of *OTU000059* and *OTU000095*.

| **OTU** | **Phylum** | **Sequence** | **bp** |
| --- | --- | --- | --- |
| *OTU000059* | Unclassified | CTTGGTCATTTAGAGGAAGTAAAAGTCGTAACAAGGTCTCCGTAGGTGAACCTGCGGAGGGATCATTTGAGAATCCGGGTGCGGCCTGGCCATGCGTTTGCGCGCTCTGACCTGCCGTGCGCCCGCCCTTCGGCCTGTGGAAGCGAGCTGTGCTTCGGCGTCCCTCCGTGACGCCGCCTTTATGCTGTCTGGCTTGTCTTGTCTGTTAAGAAAAGAGAAATCAAAACTTTCAACAAAGGATCTCTTGGCTCTCGCATCGATGAAGAACGCAGC | 273 |
| *OTU000095* | Ascomycota | CTTGGTCATTTAGAGGAAGTAAAAGTCGTAACAAGGTTTCCGTAGGTGAACCTGCGGAAGGATCATTACCGAGTTAGGGTCTTGTAGGCCCAATCTCCAACCCTGTGTATATATAATTTCGTTGCTTTGGCAGGCCTGTCTTTGACCAGACTGCCGGAGGTCCCCCGGACCGCCGGAGAGTGCCTGTCAGTAGCCCCCATTTTAAATTCGTGAATAATCGTGACGTCTGAGTTGAGATAACAAATAGTTAAAACTTTCAACAACGGATCTCTTGGTTCTGGCATCGATGAAGAACGCAGC | 300 |

**Table S6** Z-scores of various environmental factors in the rhizosphere soil of tea trees

| **Sample** | **pH** | **EC** | **OC** | **OP** | **AP** | **AK** |
| --- | --- | --- | --- | --- | --- | --- |
| QS-H1 | 2.000646 | -0.33078 | 0.455939 | -0.18459 | -1.49085 | -0.3443 |
| QS-H2 | 1.223492 | -1.14477 | 0.328615 | 0.214129 | 1.117052 | -0.50961 |
| QS-H3 | 0.992446 | -1.19665 | 0.628735 | 0.066454 | -0.27383 | -0.49133 |
| QS-H4 | 1.2655 | -1.24852 | 0.483222 | -0.65716 | -1.66471 | -0.22622 |
| QS-H5 | 0.950438 | -0.89738 | 0.649955 | -0.16983 | -0.09997 | -0.58465 |
| QS-M1 | 0.467342 | -0.3627 | 1.154701 | 1.277393 | -1.14313 | -0.97203 |
| QS-M2 | 0.488347 | -0.56221 | 1.02283 | 1.484139 | -0.88234 | -1.26228 |
| QS-M3 | 0.488347 | -1.00113 | 1.477556 | 1.631814 | -1.31699 | -1.52853 |
| QS-M4 | 0.572363 | -1.04103 | 1.39419 | 1.764722 | -0.70848 | -1.85039 |
| QS-M5 | 0.467342 | -1.44004 | 1.492714 | 1.986235 | 0.421611 | -1.39597 |
| WS-H1 | -0.45684 | 0.407394 | -1.31749 | -0.56855 | 1.464773 | 1.020097 |
| WS-H2 | -0.45684 | 0.654783 | -1.25686 | -0.55378 | 0.769332 | 1.071138 |
| WS-H3 | -0.51985 | 0.391434 | -1.23109 | -0.67192 | 0.595472 | 1.039904 |
| WS-H4 | -0.49885 | 1.305178 | -1.40389 | -0.79006 | 0.595472 | 1.045237 |
| WS-H5 | -0.49885 | 0.57099 | -1.222 | -1.02634 | 1.290913 | 0.908492 |
| WS-M1 | -1.255 | 1.081729 | -0.59145 | -0.68669 | 1.290913 | 0.809077 |
| WS-M2 | -1.31801 | 1.165522 | -0.61418 | -0.70146 | 0.856262 | 0.791936 |
| WS-M3 | -1.31801 | 1.604439 | -0.51869 | -0.87867 | -0.96927 | 0.888685 |
| WS-M4 | -1.33901 | 1.165522 | -0.41562 | -0.59809 | -0.44769 | 0.952677 |
| WS-M5 | -1.255 | 0.878232 | -0.51718 | -0.93774 | 0.595472 | 0.638051 |

**Table S7** Bray-Curtis distance statistics between samples (N = 20)

| **Sample ID** | **Abundant taxa** | **Intermediate taxa** | **Rare taxa** |
| --- | --- | --- | --- |
| QS-H1 | 0.753379 | 0.785125 | 0.871347 |
| QS-H2 | 0.689439 | 0.651961 | 0.785477 |
| QS-H3 | 0.689409 | 0.711377 | 0.800695 |
| QS-H4 | 0.633384 | 0.676317 | 0.821075 |
| QS-H5 | 0.624785 | 0.640111 | 0.7895 |
| QS-M1 | 0.629362 | 0.717031 | 0.809941 |
| QS-M2 | 0.627817 | 0.689081 | 0.806578 |
| QS-M3 | 0.585722 | 0.656973 | 0.821764 |
| QS-M4 | 0.60117 | 0.680115 | 0.810479 |
| QS-M5 | 0.593583 | 0.650755 | 0.781224 |
| WS-H1 | 0.649689 | 0.727988 | 0.803276 |
| WS-H2 | 0.603365 | 0.606433 | 0.812826 |
| WS-H3 | 0.598965 | 0.64796 | 0.783169 |
| WS-H4 | 0.748439 | 0.656166 | 0.77237 |
| WS-H5 | 0.635865 | 0.668001 | 0.820946 |
| WS-M1 | 0.621359 | 0.724547 | 0.779818 |
| WS-M2 | 0.613221 | 0.657004 | 0.81365 |
| WS-M3 | 0.611109 | 0.684562 | 0.788071 |
| WS-M4 | 0.706282 | 0.731006 | 0.819316 |
| WS-M5 | 0.587371 | 0.685237 | 0.801346 |

**Figure S1** Species stacking map based on phylum level for abundant taxa (N= 20).


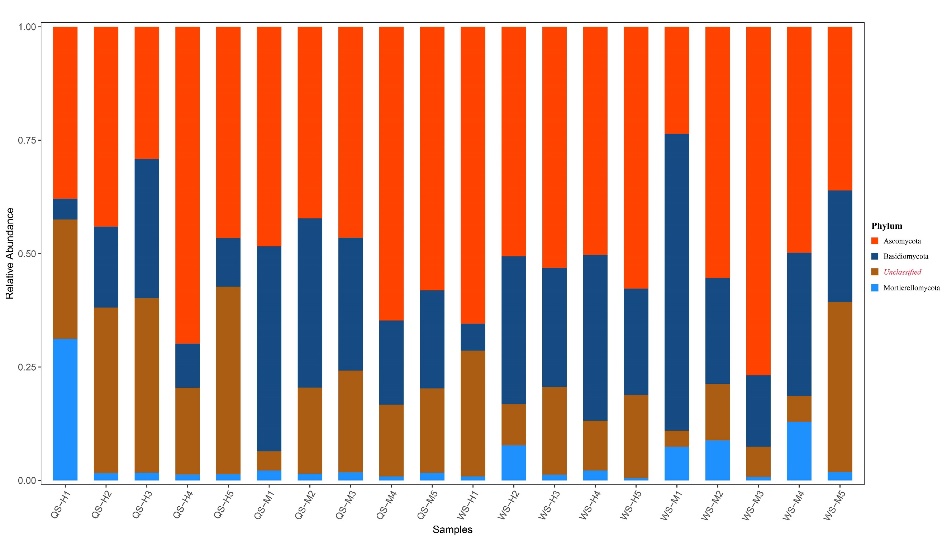


**Figure S2** Species stacking map based on phylum level for intermediate taxa (N= 20).


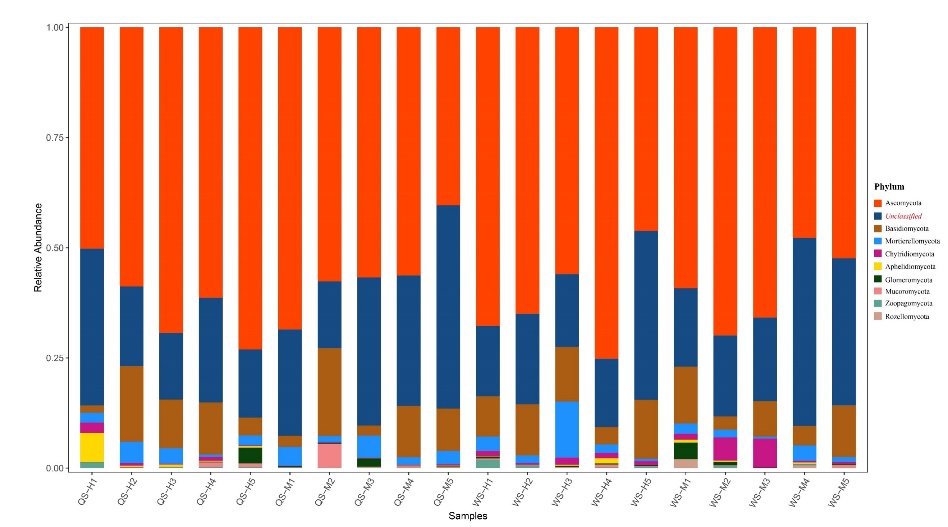


**Figure S3** Species stacking map based on phylum level for rare taxa (N= 20).


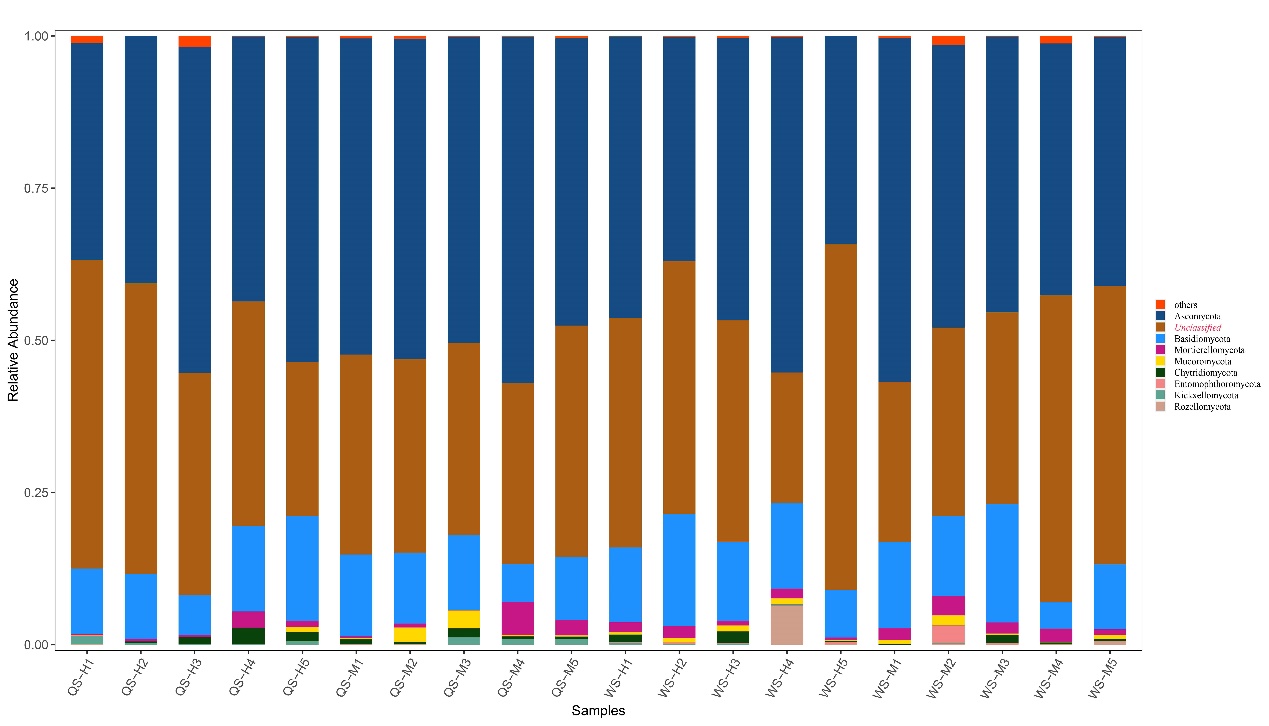


**Figure S4** Comparison between healthy (H) and diseased (M) samples α-diversity index


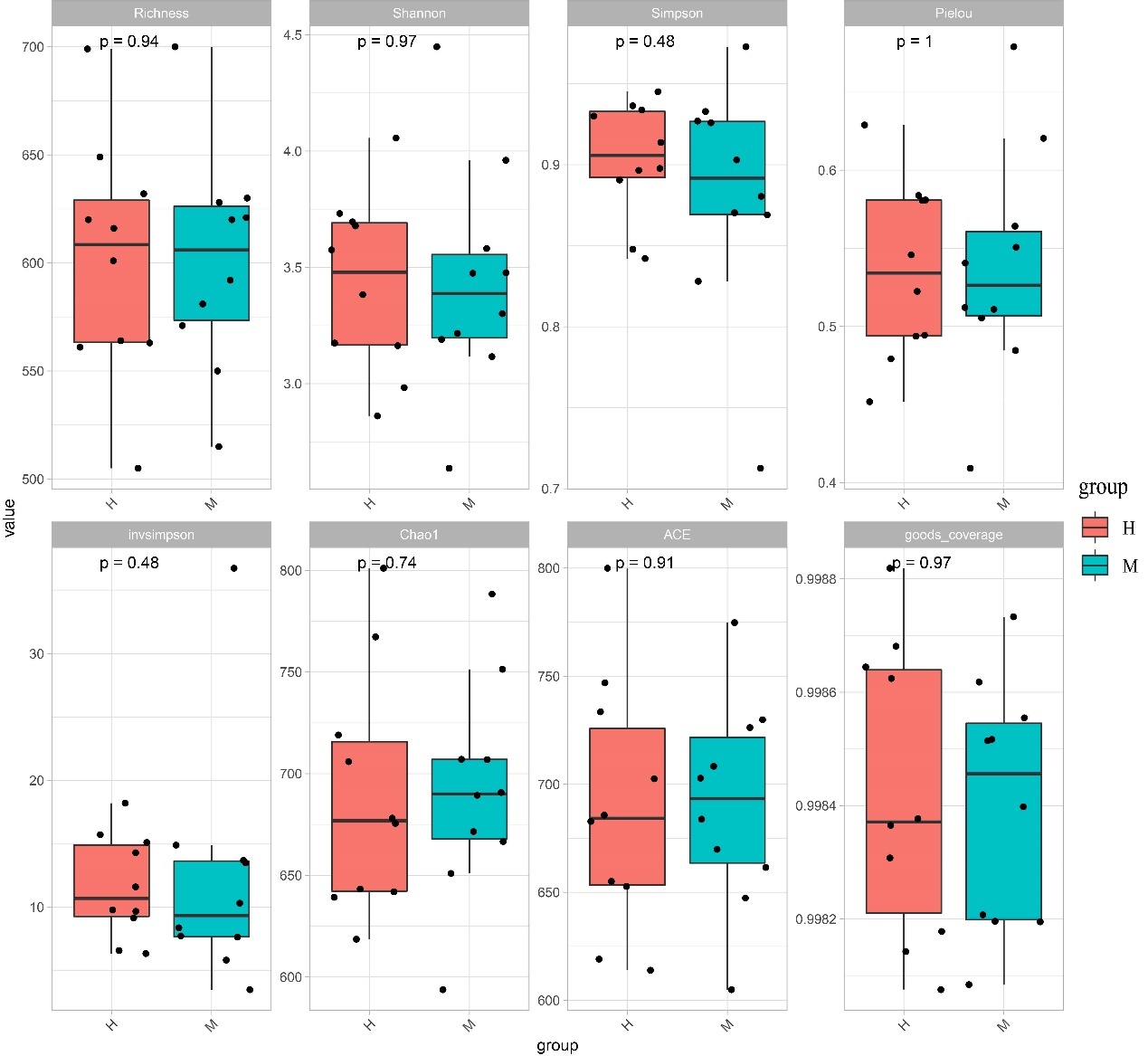


**Figure S5** Orthogonal Partial Least Squares Discriminant Analysis (OPLS-DA) of AT under the Influence of tea tree diseases based on OUT level. (a) Statistics of differences in OTU between H and M groups; (b) OPLS-DA scores for H and M groups; (c) the most extreme values (positive and negative) of three variables loaded each time are black and labeled; (d) OPLS-DA statistical model Permutation Test validation.


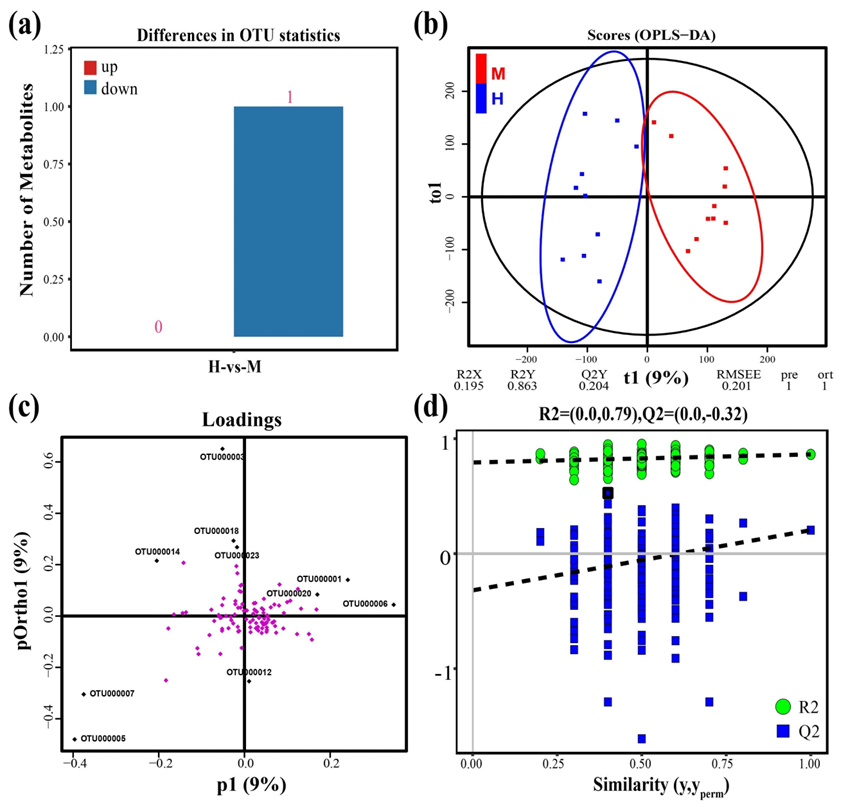


**Figure S6** Orthogonal Partial Least Squares Discriminant Analysis (OPLS-DA) of RT under the Influence of tea tree diseases based on OUT level. (a) Statistics of differences in OTU between H and M groups; (b) OPLS-DA scores for H and M groups; (c) the most extreme values (positive and negative) of three variables loaded each time are black and labeled; (d) OPLS-DA statistical model Permutation Test validation.


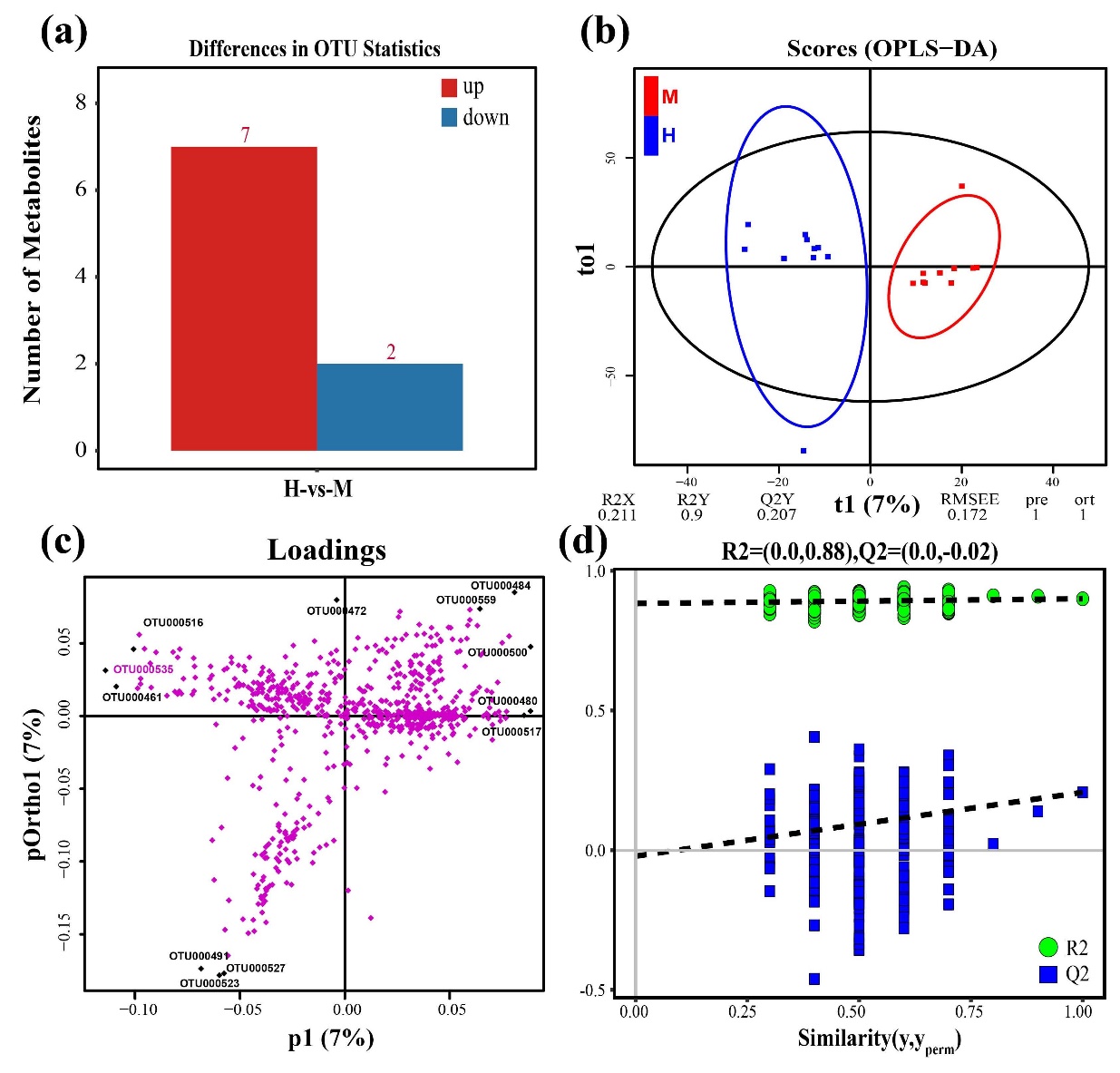


**Figure S7** Geographical map of the study areas. **(a)** The relative position of the province where the research area is located (Guizhou Province) in China; **(b)** the geographical location of the sampling area in Guizhou Province; **(c and d)** the distribution of karst landforms in the counties and districts where the sampling points are located.


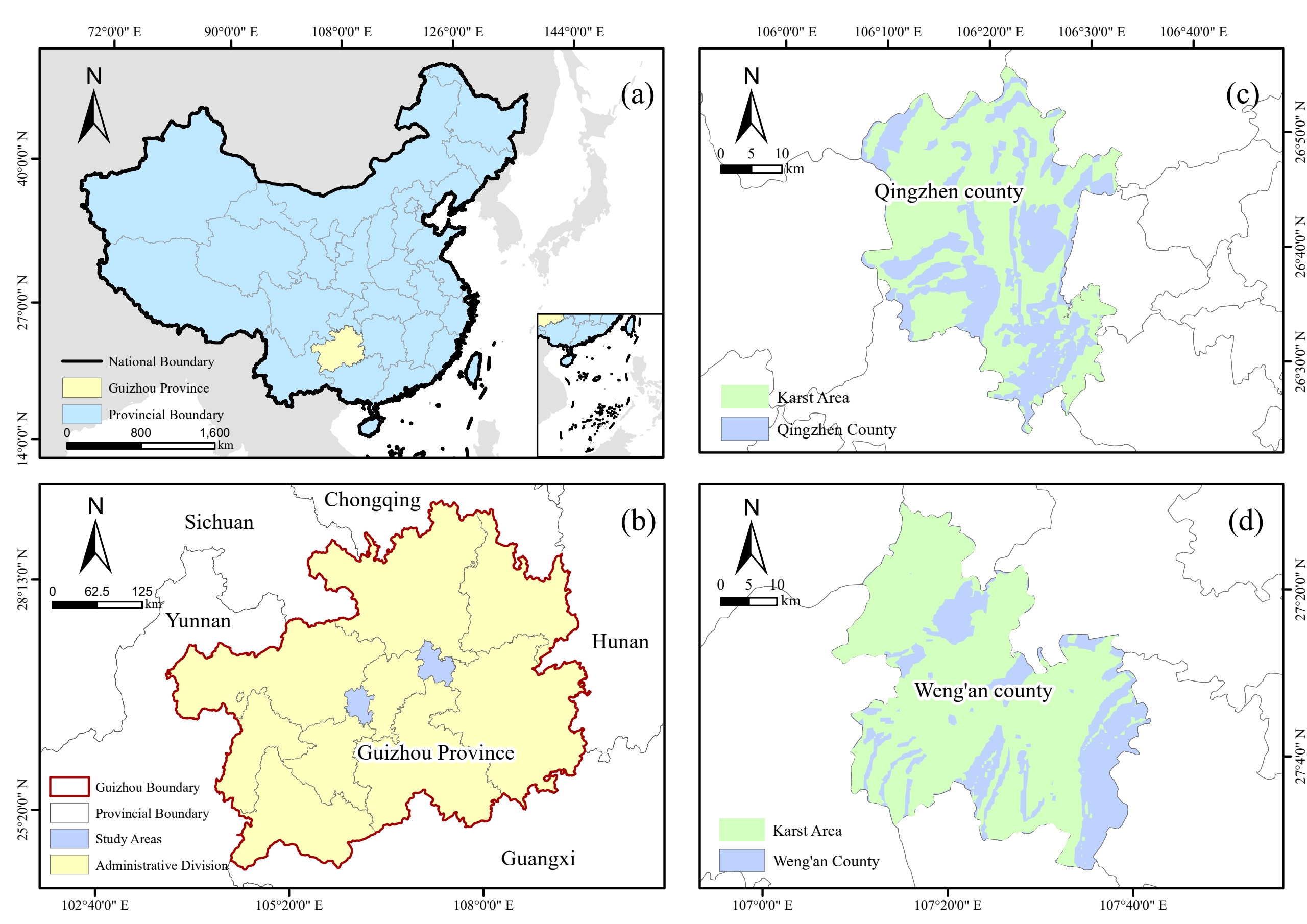

Supplement: Supplemental material — Tables S1 to S7; Fig. S1 to S7. [file spectrum.01889-24-s0001.docx]
